# Striving for clinical consensus in the management of Charcot’s neuroarthropathy

**DOI:** 10.1186/1757-1146-4-S1-O39

**Published:** 2011-05-20

**Authors:** Joseph Rogers

**Affiliations:** 1 John Morris Diabetes Centre, Launceston General Hospital, Launceston, Tasmania, 7300, Australia

## 

The management of an individual with Charcot’s Neuroarthropathy can be complex, problematic and extremely labour intensive. In order to achieve desirable clinical outcomes the management of this condition must be timely and multidisciplinary all the while keeping the patient informed of their progression throughout the treatment plan. Of the difficulties faced when implementing management of this patient group, few are more challenging than that of gaining full multidisciplinary support for the treatment plan. In an attempt to standardise the management of Charcot’s Neuroarthropathy and to overcome some of the difficulties faced, the Podiatry Department of the John Morris Diabetes Centre conducted a lengthy literature review of diagnoses and management techniques for Charcot’s Neuroarthropathy. Out of this review a treatment flowchart was developed along with a central management record to guide treatment choice and to track the patient’s progress. These tools now serve not only to promote awareness of this condition when filed in the patient record, they also enable all clinicians involved in the patients care to be aware of the progress of the disease process and management plan. These documents have now been adopted throughout many of the public podiatry services in Tasmania making the collection of larger amounts of data surrounding Charcot’s Neuroarthropathy possible, facilitating further credible research and making our treatment approach more uniform.

